# Brain white matter hyperintensity lesion characterization in 3D T_2_ fluid-attenuated inversion recovery magnetic resonance images: Shape, texture, and their correlations with potential growth

**DOI:** 10.3389/fnins.2022.1028929

**Published:** 2022-11-24

**Authors:** Chih-Ying Gwo, David C. Zhu, Rong Zhang

**Affiliations:** ^1^Department of Information Management, Chien Hsin University of Science and Technology, Taoyuan City, Taiwan; ^2^Department of Radiology, Cognitive Imaging Research Center, Michigan State University, East Lansing, MI, United States; ^3^Department of Psychology, Cognitive Imaging Research Center, Michigan State University, East Lansing, MI, United States; ^4^Department of Neurology and Internal Medicine, University of Texas Southwestern Medical Center, Dallas, TX, United States; ^5^Institute for Exercise and Environmental Medicine, Texas Health Presbyterian Hospital Dallas, Dallas, TX, United States

**Keywords:** white matter hyperintensity, 3D Zernike transformation, shape, texture, potential growth index

## Abstract

Analyses of age-related white matter hyperintensity (WMH) lesions manifested in T_2_ fluid-attenuated inversion recovery (FLAIR) magnetic resonance images (MRI) have been mostly on understanding the size and location of the WMH lesions and rarely on the morphological characterization of the lesions. This work extends our prior analyses of the morphological characteristics and texture of WMH from 2D to 3D based on 3D T_2_ FLAIR images. 3D Zernike transformation was used to characterize WMH shape; a fuzzy logic method was used to characterize the lesion texture. We then clustered 3D WMH lesions into groups based on their 3D shape and texture features. A potential growth index (PGI) to assess dynamic changes in WMH lesions was developed based on the image texture features of the WMH lesion penumbra. WMH lesions with various sizes were segmented from brain images of 32 cognitively normal older adults. The WMH lesions were divided into two groups based on their size. Analyses of Variance (ANOVAs) showed significant differences in PGI among WMH shape clusters (*P* = 1.57 × 10^–3^ for small lesions; *P* = 3.14 × 10^–2^ for large lesions). Significant differences in PGI were also found among WMH texture group clusters (*P* = 1.79 × 10^–6^). In conclusion, we presented a novel approach to characterize the morphology of 3D WMH lesions and explored the potential to assess the dynamic morphological changes of WMH lesions using PGI.

## Introduction

White matter hyperintensities (WMH) on T_2_ fluid-attenuated inversion recovery (FLAIR) magnetic resonance brain images (MRI) are commonly observed in older adults over 65 years old with a prevalence rate of ∼ 60–80% in the general population (De Leeuw et al., 2001; Wen and Sachdev, 2004). WMH lesions are even more extensive in those with vascular or Alzheimer’s disease (AD) type of dementia when compared with cognitively normal older adults, suggesting its role in dementia pathogenesis and neurocognitive dysfunction (Bombois et al., 2007; Kloppenborg et al., 2014; Lee et al., 2016). WMH is also frequently observed in patients with multiple sclerosis (MS) (Loizou et al., 2015; Newton et al., 2017). Qualitative and quantitative WMH characterization has been used as a biomarker to assist cerebral small vessel disease diagnosis and to assess treatment effects (Wardlaw et al., 2013). The pathogenic mechanisms of WMH are not well understood, and have been attributed to brain hypoperfusion, white matter demyelization, or both (Greenberg, 2006; Wardlaw et al., 2013). Furthermore, periventricular and subcortical deep WMHs may have different pathogenic mechanisms (Schmidt et al., 2011; Poels et al., 2012; Tseng et al., 2013). The commonly used methods for WMH quantification are to measure its regional or total volume (i.e., the sum of WMH voxel size) within the whole brain based on image tissue segmentation algorithms (DeCarli et al., 2005; Wardlaw et al., 2013). This method, however, neglects entirely the typological or morphological features of WMH lesions which may have important clinical significance as demonstrated in recent studies in patients with MS (Loizou et al., 2015; Newton et al., 2017). In this regard, imaging processing using deep learning may reveal image patterns related to disease progression (Zeng et al., 2021; Li et al., 2022). However, this approach has limitations in that the unique typological or morphological features of WMH and its underlying neurobiological mechanisms cannot be characterized.

White matter hyperintensities shape is a basic morphological feature which can be derived from high-resolution T_2_ FLAIR images after tissue segmentation. Shape feature extraction, recognition, and classification can be implemented either in the original or the transformed image space (Khotanzad and Hong, 1990; Mikolajczyk et al., 2003; Carmichael and Hebert, 2004; Tahmasbi et al., 2011). The analysis of 3D shape has been widely applied in the fields of image processing and pattern recognition, such as terrain matching (Rodriguez and Aggarwal, 1990), object retrieval (Körtgen et al., 2003; Novotni and Klein, 2004), anatomical structure analysis (Gerig et al., 2001; Styner et al., 2005; Terriberry et al., 2005; Luders et al., 2006; Gerardin et al., 2009; Shen et al., 2012; Wachinger et al., 2015; Makkinejad et al., 2019), and protein structural similarity retrieval (Mak et al., 2008; Sael et al., 2008). In general, the feature vectors of 3D shapes are first extracted. Then the similarity between the vectors is indexed for comparison, clustering, and recognition. The feature representation of 3D shape is to transform the original space of 3D objects to a high-dimensional feature vector space while preserving the shape information. The resulting feature vector (also known as a shape descriptor) can be used to characterize the unique shape of an object. In (Zhang et al., 2007), the computational techniques used for obtaining shape descriptors were comprehensively reviewed, and categorizations of the approaches were also provided. A shape descriptor, in general, needs to be able to characterize both the global shape contour and the regional topological details (Deng et al., 2016). Additionally, in order to assess the reliability and the accuracy of the descriptor, the descriptor must be able to reconstruct as close to the original object as possible. Due to the complexity of 3D shape feature extraction and the computational instability of numerical feature values, low-order 3D descriptors of objects, especially with the voxel-based approach, were commonly found in current literature (Novotni and Klein, 2004; Venkatraman et al., 2009a). Although a low-order shape descriptor may provide sufficient information for classification of objects at a coarse level, higher-order shape features are required to differentiate subtle regional topological differences in objects with fine structures. Therefore, choosing an appropriate order of shape descriptor is crucial to represent a 3D shape with different morphological features.

Based on literature, three categories of algorithms have applied to study the shapes of 3D objects: (1) surface-based methods using spherical harmonics as the basis functions (Kelemen et al., 1999; Styner et al., 2006), (2) voxel-based methods based on 3D Fourier (Vranic and Saupe, 2001) and Zernike transforms (Novotni and Klein, 2003), and (3) spectrum-based methods by solving the eigenvalues of 2D and 3D Laplace-Beltrami operators on triangular (boundary) and tetrahedral (volume) meshes (Reuter et al., 2006; Lian et al., 2013). All these methods theoretically can characterize 3D object shapes with high fidelity and have been applied to characterize brain structures (Joshi et al., 1997; Gerig et al., 2001; Styner et al., 2006; Wachinger et al., 2015, 2016). However, the surface-based methods are only suitable for smooth shapes with spherical topology, and cannot characterize 3D structures with holes or torus-like surfaces. Spectrum-based methods are isometry invariance, but cannot easily distinguish resembling objects (Reuter et al., 2006; Lian et al., 2013). Among the methods used for shape feature extraction, the voxel-based 3D Zernike transformation has its unique advantages. In contrast to surface-based methods (Zhang et al., 2007), 3D Zernike transform can characterize holes and torus. Compared to the surface-based method based on spherical harmonics, the 3D Zernike transformation combines spherical harmonics with radial polynomials to produce a more compact representation and requires fewer expansion orders (Venkatraman et al., 2009a). The advantages of 3D Zernike descriptor over spherical harmonics have been demonstrated in the benchmark studies involving image retrieval for general 3D objects (Novotni and Klein, 2004) and protein molecules with similar global structures (Sael et al., 2008). Furthermore, Zernike transform is rotational invariance in space, but Fourier transform is not which may lead to the dependence of the derived shape features on the orientation of original objects.

Based on the orthogonality of 2D Zernike moments (Teague, 1980), Canterakis generalized the classical 2D Zernike polynomials to 3D and derived 3D Zernike polynomials and moments. With this theory, Novotni and Klein developed methods for computing the Zernike moments and object reconstruction (Novotni and Klein, 2003, 2004). Similar to the 2D Zernike moments, the magnitudes of complex 3D Zernike moments, named the Zernike descriptor, is rotational invariant.

The 3D Zernike descriptors have been successfully applied to protein structural similarity retrieval (Mak et al., 2008; Sael et al., 2008), protein-protein docking using region-based (Venkatraman et al., 2009b), terrain matching (Ye and Chen, 2012), and amphetamine-type stimulant (ATS) drugs identification (Pratama et al., 2015). The maximum order of 3D Zernike moments used in most, if not all, studies is below 30 and can only represent the rough shape features of 3D objects because the existing computation method developed by Novotni and Klein is time-consuming and computationally instable for calculating higher orders of Zernike moments (Zhang et al., 2007). Recently, we have proposed a new algorithm to calculate high order 3D Zernike moments to characterize objects which have fine structures (Deng and Gwo, 2020).

Texture is also a basic feature of the surface appearance of an object, and one of the important morphological features of images. Textured surfaces are the core of human vision because they are important visual cues about surface characteristics. Texture information is used to identify objects and understand the pre-attentional vision of the scene (Depeursinge et al., 2014). Texture analysis has received widespread attention because of its important role in the field of computer vision and pattern recognition, including facial analysis, industrial inspection, satellite or aerial image analysis, biomedical image analysis, and biometrics object recognition (Depeursinge et al., 2017; Liu et al., 2017). Image texture can be assessed using several quantitative approaches such as structural, spectral transformation, modeling, or statistics-based approaches (Bharati et al., 2004). For image texture analysis, the statistical approaches have the benefits of being rotational, size, and translational invariant in the feature vector spaces. Furthermore, these methods can characterize image intensity distributions directly (Bharati et al., 2004; Castellano et al., 2004). In addition, they require fewer a priori model assumptions, such as basic symbolic image elements and repeated image patterns (Castellano et al., 2004). We used a statistical method based on fuzzy logic to construct the image intensity histogram of WMH lesions, and then cluster lesion features into several groups. Significant differences in the intensity of lesions can be observed in the resulting groups (Gwo et al., 2019). However, the concept of three-dimensional texture is rarely used because textures that exist in more than two dimensions cannot be fully visualized by humans. Computer graphics only provide virtual navigation in multi–planar rendering or translucent visualization and allow observation of two-dimensional projections of opaque textures (Toriwaki and Yoshida, 2009). Depeursinge et al. provided a good review of the challenges and opportunities of 3D texture analysis in biomedical imaging (Depeursinge et al., 2014).

White matter hyperintensities morphological characteristics such as the size, shape, and image texture may change with time which may reflect the progression of underlying dynamic pathophysiological process (Novotni and Klein, 2003; Lian et al., 2013). In this regard, recent studies have shown that the immediate surrounding areas of the defined WMH lesions may be at risk for further tissue damage and conversion to lesions (Reuter et al., 2006; Wachinger et al., 2016). These areas are classified as WMH penumbras (Wachinger et al., 2016). To characterize WMH lesions as well as their penumbras, we developed a seed-based region-growing algorithm to characterize 2D WMH boundaries to explore the potential growth of WMH lesions. We defined this specific WMH boundary characteristic as potential growth index (PGI) and observed that both shape and texture characteristics of 2D WMH are related to PGI (Gwo et al., 2019).

The characterization and quantification of the shape and texture of 3D WMH lesions have not been previously attempted. In this work, we extended 2D to high-order 3D Zernike transform to study the shape of 3D WMH and applied fuzzy logic to the intensity histogram of 3D WMH lesions for texture feature extraction. We also explored the potential growth index to predict of future tissue damage surrounding the 3D WMH lesion, that is, the WMH penumbra. Finally, we evaluated 3D potential growth differences among different lesion shape categories and texture clusters.

## Methods and results

### Magnetic resonance images acquisition

Full-brain 3D T_2_ FLAIR images with voxel size of 1 × 1 × 1 mm^3^ were collected on a GE Discovery MR 750W 3T MRI scanner (GE Healthcare, Waukesha, WI) with the following parameters: sagittal, time of echo (TE) = 115 ms, time of repetition (TR) = 6.8 s, time of inversion (TI) = 1828 ms, echo train length = 200, receiver bandwidth = 41.67 kHz, fat saturation on, field of view (FOV) = 25.6 × 25.6 cm, slice thickness = 1 mm, number of slices = 176, acquisition matrix size = 256 × 256. All subjects signed informed consent approved by the Institutional Review Boards of the UT Southwestern Medical Center and Texas Health Presbyterian Hospital of Dallas. Thirty-two T_2_ FLAIR brain image datasets (15 male, 17 female, 66.7 ± 6.0 years old and normal cognition), which contained clearly identifiable white matter hyperintensity (WMH) lesions with various sizes, were selected from an on-going HIPAC clinical trial (NCT03354143). Inclusion criteria: (1) age 55–79 years; (2) Mini-Mental State Exam (MMSE) > 26 to exclude dementia; (3) normotensive subjects and patiens with hypertension Exclusion criteria: (1) severe cerebrovascular disease such as stroke, transient ischemic attack, traumatic brain injury; (2) clinical diagnosis of dementia or other neurodegenerative diseases; (3) severe depression or other psychopathology; (4) unstable heart disease; (5) chronic kidney diseases with GFR < 40 ml/min; (6) orthostatic hypotension; (7) history of significant autoimmune disorders; (8) history of drug or alcohol abuse within the last 2 years; (9) uncontrolled diabetes mellitus; (10) obstructive sleep apnea; (11) regularly smoking cigarette within the past year; (12) severe obesity with BMI ≥ 45; (13) carotid stent or severe stenosis (> 50%); (14) pacemaker or other medical device of metal that precludes performing MRI; (15) history B12 deficiency or hypothyroidismT_2_ FLAIR Image Segmentation.

**FIGURE 1:**
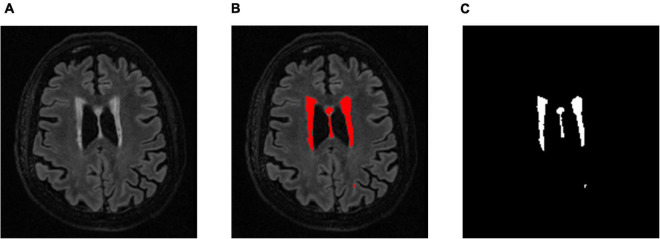
**(A)** An example a slice of T_2_ FLAIR image volume from one subject showing multiple white matter hyperintensity (WMH) lesions; **(B)** the results of WMH segmentation using the lesion prediction algorithm (LPA) showing in red, and **(C)** a WMH binary mask after tissue segmentation, which was used to form the 3D structure of WMH for feature extraction.

T_2_ FLAIR WMH regions were segmented on each 3D image volume through the lesion prediction algorithm (LPA) implemented in the Lesion Segmentation Toolbox (LST) version 2.0.12 for Statistical Parametric Mapping (SPM12). In LPA, the algorithm is trained using a logistic regression model on T_2_ FLAIR brain images from 53 MS patients with severe lesion patterns. LPA was also validated in other patient populations such as older adults with diabetes (Styner et al., 2005). The fitness of a new T_2_ FLAIR brain image to this model provides an estimate of lesion probability for each voxel in the image. In this study, we used a threshold of 0.5, as suggested by LST, on the obtained lesion probability maps to identify WMH regions. The segmentation accuracy was further verified through visual inspection. Figure 1 shows an example of the segmentation.

### Lesion size distribution

**FIGURE 2:**
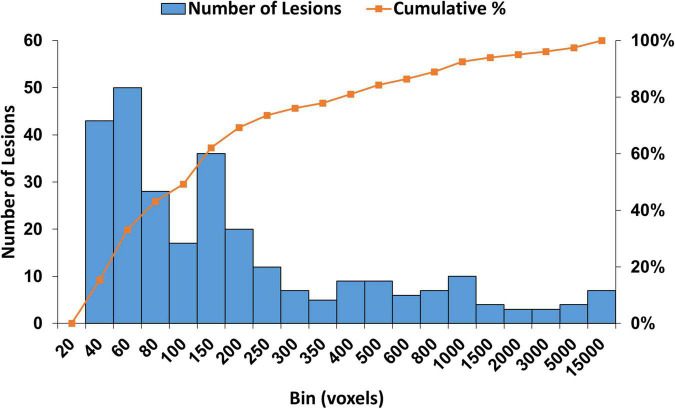
The histogram of WMH3D lesion size distribution for all subjects. The cumulative lesion size distribution is shown in percentage on the right vertical axis.

We extracted the WMH3D lesions greater than 30 mm^3^ in each subject and obtained a total number of 280 lesions. The lesion size distributions of all subjects are shown in Figure 2. The figure shows a wide range of lesion sizes and most of the lesions are relatively small. To explore whether the shape of WMH3D is related to its potential growth, the volume of all WMH3D is required to normalize to the same size. However, the volume scaling process can add or lose 3D shape details, and more so when the size distribution of WMH3D has a wide range as in our subjects (Figure 2). To reduce this scaling issue, this study divided the 280 WMH3D lesions to two groups based on their size. Group *S_s_* had lesion size smaller than or equal to 250 voxels, and Group *S_l_* had lesions larger than 250 voxels The group division generated 206 lesions for *S*_*s*_ and 74 lesions for *S_l_*. To understand the anatomical distribution of the 280 WMH3D lesions, we classified the WMH clusters within or adjacent to the ventricle borders with a 3-mm thickness as periventricular WMH, and the rest as deep WMH. We found that 84.6% of the WMH clusters were at the periventricular region, and they tended to be relatively large with a volume size of 587.3 ± 1660.5 (mm^3^) with a range of 31–12409 mm^3^. The deep WMH clusters tended to be small with a volume size of 72.0 ± 44.4 (mm^3^), with a range of 31–256 mm^3^.

**FIGURE 3:**
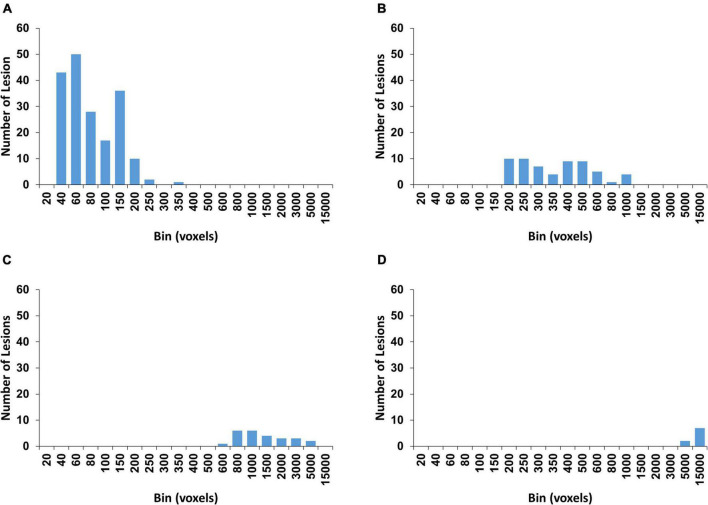
The four clusters with the histogram of the WMH3D volume size distribution are determined by gap statistic. The numbers of WMH3D in each cluster **(A**–**D)** are 187, 59, 25, and 9, respectively.

The rationale of using 250 voxels as the cut-off for the lesion groups was based on both volume and shape characteristics of the lesions. All extracted WMH3D lesions were positioned to 150 × 150 × 150 cubes with their centroids at the centers. Then 3D Zernike transform with an order of 150 was applied to the cubes to obtain the corresponding shape descriptors, and then a K-mean algorithm was used to cluster all 280 lesions into four clusters which are determined by the gap statistic (GAP, described in the later section) (Tibshirani et al., 2001). This process assisted in finding a size classification based on both the volume and shape of the lesion to reduce the influence of the shape changes caused by volume normalization on subsequent analyses. The size distribution results of GAP clustering are shown in Figure 3. The cluster shown in Figure 3A contains 187 lesions. The size of 250 voxels is a proper cutoff and thus was chosen to divide 280 lesions by their sizes into two groups.

### WMH3D shape feature extraction and classification

#### WMH3D shape feature extraction using 3D Zernike transformation

The 2D Zernike transformation is based on the Zernike polynomials defined on the unit disc *D*. This transformation has been extensively applied to imaging shape feature extraction and pattern recognition (Papakostas et al., 2007; Wee and Paramesran, 2007; Gwo et al., 2019). The coefficients of the Zernike polynomial expansion of an object are called Zernike moments (ZMs). The magnitude of the ZMs, which is also named as the Zernike descriptor, is rotational invariant and represents the shape features of the analyzed objects. To define the 3D version of Zernike polynomials, the unit disc *D* is replaced by a unit ball *B*. Every point (*x*,*y*,*z*) in the unit ball *B* can be represented by a spherical coordinates (*r*, *θ*, *ϕ*) as shown in Eq. 1,


(1) $$Z_{n\,m}=\frac{n+1}{\pi }\,\int_{0}^{2\,\pi }\int_{0}^{1}f\,\left(r,\theta \right)\,V_{n\,m}^{*}\,\left(r,\theta \right)\,r\,dr\,d\theta ,\left|r\right|\leq 1,$$

where


(2) $$\begin{array}{ccc} r & = & \sqrt{x^{2}+y^{2}+z^{2}}\\ \theta & = & \cos^{-1}\frac{z}{r}\\ \phi & = & \sin^{-1}\frac{y}{\sqrt{x^{2}+y^{2}}} \end{array}$$

Canterakis introduced the first algorithm to calculate 3D Zernike moments (3DZMs) (Canterakis, 1999), where the 3DZMs were expressed as the linear combination of geometric moments. These 3DZMs were later described as shape descriptors for shape retrieval (Novotni and Klein, 2004). Canterakis’ algorithm has been applied to terrain matching (Wang et al., 2018a,b) and protein–protein interface prediction (Daberdaku and Ferrari, 2018). However, Canterakis’ algorithm could only be used to compute ZM up to the order of 25, due to computational demand and instability. Hosny et al. introduced a fast algorithm using eight ways of (anti-)symmetries (Hosny and Hafez, 2012). To overcome the limitations on computational efficiency and the maximum ZM order that can be computed reliably in previous algorithms, Deng and Gwo proposed a new algorithm based on a recursive approach to calculate 3D Zernike radial polynomials, as described in Eqs 10–13 (Deng and Gwo, 2020). The algorithm used to calculate the 3D Zernike polynomial is briefly described below.

The 3D Zernike polynomial $V_{n\,\ell }^{m}\,\left(r,\theta ,\phi \right)$ is defined as the multiplication of spherical harmonic $Y_{\ell }^{m}\,\left(\theta ,\phi \right)$ and radial polynomial *R*_*n*ℓ_ (*r*)as below:


(3) $$V_{n\,\ell }^{m}\,\left(r,\theta ,\phi \right)=Y_{\ell }^{m}\,\left(\theta ,\phi \right)\,R_{n\,\ell }\,\left(r\right)$$

$Y_{\ell }^{m}\,\left(\theta ,\phi \right)$ and *R*_*n*ℓ_ (*r*) are computed separately. The spherical harmonic $Y_{\ell }^{m}\,\left(\theta ,\phi \right)$ of degree ℓ with order *m* is given by


(4) $$Y_{\ell }^{m}\,\left(\theta ,\phi \right)=\left\{ \begin{array}{cc} K_{\ell }^{m}\,P_{\ell }^{m}\,\left(\cos\theta \right)\,\cos m\,\phi \,\text{if}\,m\geq 0 & \\ K_{\ell }^{m}\,P_{\ell }^{m}\,\left(\cos\theta \right)\,\sin m\,\phi \;\text{otherwise} & \end{array}\right.$$

where $P_{\ell }^{m}\,\left(\cdot \right)$is the associated Legendre polynomial of degree ℓ, given by


(5) $$P_{\ell }^{m}\,\left(x\right)={\left(-1\right)}^{m}\,{\left(1-x^{2}\right)}^{\frac{m}{2}}\,\frac{d^{m}}{d\,x^{m}}\,\left(P_{\ell }\,\left(x\right)\right)$$

and $K_{\ell }^{m}$ is the normalizing factor given by


(6) $$K_{\ell }^{m}={\left(-1\right)}^{m}\sqrt{\frac{\in_{m}\left(2\,\ell +1\right)\,\left(\ell -m\right)!}{\left(\ell +m\right)!}}\text{where}\in_{m}=\left\{ \begin{array}{cc} 1 & \text{if}\,m\neq 0\\ 2 & \text{oterwise} \end{array}\right.$$

Let $\tilde{P_{\ell }^{m}}\,\left(\cos\theta \right)=K_{\ell }^{m}\,P_{\ell }^{m}\,\left(\cos\theta \right)$ be the normalized associated Legendre polynomial. Then Eq. 4 is simplified to


(7) $$Y_{\ell }^{m}\,\left(\theta ,\phi \right)=\left\{ \begin{array}{cc} \tilde{P_{\ell }^{m}}\,\left(\cos\theta \right)\,\cos m\,\phi \,\text{if}\,m\geq 0 & \\ \tilde{P_{\ell }^{m}}\,\left(\cos\theta \right)\,\sin m\,\phi \,\text{otherwise} & \end{array}\right.$$

The spherical harmonics $Y_{\ell }^{m}\,\left(\cdot \right)$form an orthonormal basis for the Hilbert space *L*^2^(*S*^2^) of the square-integrable functions over the unit sphere *S*^2^. For any function *f* of *L*^2^(S^2^), *f* can be expressed as in Eq. 8 (Szegõ, 1939):


(8) $$f\,\left(\theta ,\phi \right)=\sum_{\ell =0}^{\infty }\sum_{\text{m}=-\ell }^{\ell }C_{\ell }^{m}\,Y_{\ell }^{m}\,\left(\theta ,\phi \right)$$

where $C_{\ell }^{m}$ are the coefficients; ℓ is a non-negative integer; *m* is an integer with |*m*| ℓ. The computation procedures of $\tilde{P_{\ell }^{m}}\,\left(\cos\theta \right)$ for degree ℓ ≤ ℓ_*max*_ are summarized to the following (Szegõ, 1939; Deng and Gwo, 2018a):

1. Initialize $\tilde{P_{0}^{0}}\,\left(\cos\theta \right)=\sqrt{\frac{1}{4\,\pi }}$, which is the normalizing factor for volumetric integration. Then iteratively calculate the following:
2. $\tilde{P_{\ell }^{\ell }}\,\left(\cos\theta \right)={\text{C}}_{3}\,\sin\theta \,\tilde{P_{\ell -1}^{\ell -1}}\,\left(\cos\theta \right)$ for ℓ1,2,3,…,ℓ_*max*_
3.
$\tilde{P_{\ell }^{\ell -1}}\,\left(\cos\theta \right)={\text{C}}_{1}\,\cos\theta \,\tilde{P_{\ell -1}^{\ell -1}}\,\left(\cos\theta \right)$

4.
Pℓm ~(cosθ)=C1cosθPℓ-1m ~(cosθ)-C2Pℓ-2 m~(cosθ)

(9) $$\begin{array}{ccc} form & = & 0,1,..,\ell -2\\ \text{C} & = & \sqrt{\frac{2\,\ell +1}{\left(\ell +m\right)\,\left(\ell -m\right)}},{\text{C}}_{1}=\text{C}\,\sqrt{2\,\ell -1}\\ {\text{C}}_{2} & = & \text{C}\sqrt{\frac{\left(\ell +m-1\right)\,\left(\ell -m-1\right)}{2\,\ell -3}},{\text{C}}_{3}=\sqrt{\frac{2\,\ell +1}{2\,\ell }} \end{array}$$

For a Zernike polynomial order *n* (a non-negative integer), the integer ℓ above needs to ≤ *n* and *n* − ℓ = even, and the integer *m* above needs to |*m*| ≤ ℓ.

The 3D Zernike radial polynomial *R*_*n*ℓ_(*r*) in Eq. 3 was originally given in terms of Jacobi polynomials as described in (Szegõ, 1939), but different calculation methods of 3D Zernike radial polynomial have been proposed (Deng and Gwo, 2020). In our work, the *R*_*nℓ*_ is computed recursively, similar to the Kintner’s *P*-method in the case of 2D Zernike polynomials (Kintner, 1976; Deng and Gwo, 2018b), and is presented in Eq. 10.


(10) $$R_{n\,\ell }\,\left(r\right)=\left(K_{1}\,r^{2}+K_{2}\right)\,R_{n-2,\ell }\,\left(r\right)+K_{3}\,R_{n-4,\ell }\,\left(r\right)f\,o\,r\,n=\ell +4,\ell +6,\ldots ,n_{m\,a\,x}$$

where the coefficients *K_i_* are given by the following,


(11) $$\begin{array}{c} k_{0}=\left(n-\ell \right)\,\left(n+\ell +1\right)\,\left(2\,n-3\right)\\ k_{1}=\left(2\,n-1\right)\,\left(2\,n+1\right)\,\left(2\,n-3\right)\\ k_{2}=\left(-2\,n+1\right)\,\left(\frac{4\,\ell^{2}+4\,\ell +1}{2}\right)-\frac{k_{1}}{2}\\ k_{3}=-\left(n-\ell -2\right)\,\left(n+\ell +1\right)\,\left(2\,n+1\right)\\ K_{1}=\frac{k_{1}}{k_{0}},K_{2}=\frac{k_{2}}{k_{0}},K_{3}=\frac{k_{3}}{k_{0}} \end{array}$$

For this recursive formula, the following initial equalities are also required:


(12) $$R_{n\,n}\,\left(r\right)=r^{n}\,f\,o\,r\,n\,0,1,2,$$

and


(13) $$R_{n,n-2}\,\left(r\right)=\left(n+\frac{1}{2}\right)\,r^{n}-\left(n-\frac{1}{2}\right)\,r^{n-2}\,f\,o\,r\,n=2,3,4,\ldots$$

Let *f* (*r*, *θ*, *ϕ*) be a 3D image function within the unit ball *B*. The 3DZM ${\text{Z}}_{n\,\ell }^{m}$ can be regarded as the inner product of the image function *f* (*r*, *θ*, *ϕ*) with the basis function $V_{n\,\ell }^{m}\,\left(r,\theta ,\phi \right)$ (Deng and Gwo, 2020), and can be described as


(14) $$Z_{n\ell }^{m}=(2n+3)\underset{(r,\theta ,\phi )\in B}{∭}f(r,\theta ,\phi )V_{n\ell }^{m}(r,\theta ,\phi )r^{2}\sin\theta drd\theta d\phi$$

Each moment within Order *n* corresponds to a (2ℓ+1)-dimensional vector ${\vec{Z}}_{n\ell }$ as


(15) $$\vec{Z}=\left(Z_{n\,\ell }^{-\ell },Z_{n\,\ell }^{-\ell +1},\cdots ,Z_{n\,\ell }^{0},\cdots ,Z_{n\,\ell }^{\ell -1},Z_{n\,\ell }^{\ell }\right)$$

The *l*^2^-norm of ${\vec{Z}}_{n\ell }$, denoted by


(16) $$||\vec{Z}||=\sqrt{\sum_{m=-\ell }^{\ell }{\left|Z_{n\,\ell }^{m}\right|}^{2}}$$

is rotation invariant, and can be used as the 3D shape descriptor (or Zernike descriptor) of a 3D object. The total number of 3DZMs and the dimension of Zernike descriptor for an expansion up to order *n* are given by Eqs 17, 18, respectively:


(17) $$\mathrm{Number}\,\mathrm{of}\mathrm{3DZMs}=\sum_{i\,0}^{n}\left\lfloor \frac{{\left(i+2\right)}^{2}}{4}\right\rfloor$$

(18) $$\mathrm{Dimension}\,\mathrm{of}\,\mathrm{Zernike}\,\mathrm{descriptor}=\left\{ \begin{array}{cc} {\left(\frac{n+2}{2}\right)}^{2}\;\mathrm{if}\,\mathrm{order}\,n\,\mathrm{is}\,\mathrm{even} & \\ \frac{\left(n+3\right)\,\left(n+1\right)}{4}\,\mathrm{if}\,\mathrm{order}\,n\,\mathrm{is}\,\mathrm{odd} & \end{array}\right.$$

The image object function *f* can be reconstructed with ZM order *M* as *f_M_* below:


(19) $$f_{M}\,\left(r,\theta ,\phi \right)=\sum_{n\,0}^{M}\sum_{\ell }\sum_{m-\ell }^{\ell }Z_{n\,\ell }^{m}\,V_{n\,\ell }^{m}\,\left(r,\theta ,\phi \right)$$

When *M* is large enough, the function *f_M_* can be used to approximate the original image *f* (Deng and Gwo, 2018b). For a binary shape with the background represented by 0, the error rate ℰ_*r*_ between the original image *f* and the reconstructed *f_M_* can be calculated by


(20) ℰr=∑∀(x,y,z)(f⁢(x,y,z)⊕fM⁢(x,y,z))∑∀(x,y,z)f⁢(x,y,z) where⁢fM⁢(x,y,z)={1⁢if⁢fM⁢(x,y,z)≥0.50 ⁢otherwise                   

where ⊕ is exclusive disjunction and *f* (*x*,*y*,*z*) 0 or 1. Based on the error rate ℰ_*r*_, an appropriate ZM order M can be chosen.

Overall, the calculation of 3D Zernike moments is summarized as follows: First, the normalized associated polynomial $\tilde{P_{\ell }^{m}}\,\left(\cos\theta \right)$ of the spherical harmonic function is calculated using Eq. 9. Second, the 3D Zernike radial polynomial *R*_*nℓ*_ is recursively calculated using Eqs 10–13. Then, the 3D Zernike polynomials can be obtained by Eq. 3. Finally, Eq. 14 is used to generate 3D Zernike moments. The 3D spherical harmonics and Radial polynomials are illustrated in Supplementary Figure 1.

For 3D Zernike Transformation of WMH, all lesions were linearly rescaled at the ratio of $\sqrt[{3}]{v\prime /v}$ in three dimensions, and the intensity of the resulting voxel was calculated by tricubic interpolation (Arata, 1995), where *v* was the original volume size and the *v*′ was the volume scaled to. Due to blurring effect in scaling, an appropriate intensity threshold was then chosen so that the scaled volume is closest to *v*′. To compare the Zernike descriptors at the same scale, the lesions in Group *S*_*s*_ are normalized to about 80 voxels within a 36 × 36 × 36 cube, with the centroid at the center of the cube. Similarly, the lesions in Group *S*_*l*_ are normalized to about 1500 voxels within a 90 × 90 × 90 cube. These two numbers, 80 and 1500, are medium size in their own lesion groups.

As an example, the effect of ZM order on the Zernike transformation of two WMH3D lesions and their reconstruction accuracy is shown in Supplementary Figure 2.

**FIGURE 4:**
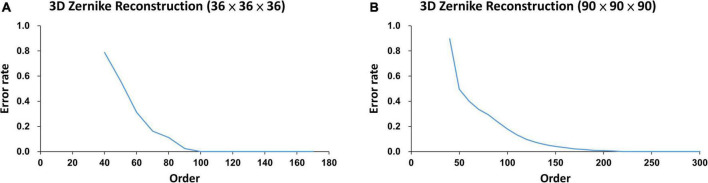
The error rates of two lesions calculated from two different normalized sizes at different Zernike reconstruction orders: **(A)** the error rate of an 80-voxel lesion in a 36 × 36 × 36 voxel cube approached zero at order around 100, and **(B)** the error rate of a 1500-voxel lesion in a 90 × 90 × 90 voxel cube approached zero at order around 250.

As shown in Supplementary Figure 2 qualitatively and in Figure 4 quantitatively, the order of the Zernike transformation needs to be large enough to preserve the original shape details.

**FIGURE 5:**
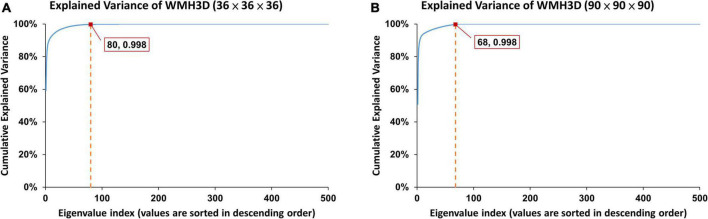
To maintain 99.8% variance of the two lesion size groups, **(A)** the first 80 principal components and **(B)** the first 68 principal components were chosen for group *S_s_* and group *S_l_*, respectively.

As illustrated in Figure 4, the error rates of WMH3D lesion reconstruction, ℰ_*r*_, would decrease with the reconstruction order. The 3D Zernike transformation with order of 100 for Group *S_s_* and then 250 for Group *S_l_*, were applied to the voxel cubes containing the size-normalized lesions. The error rates of group *S_s_* and *S_l_* were 7.3 × 10^–4^± 3.4 × 10^–3^ and 7.2 × 10^–4^± 1.3 × 10^–3^, respectively. Zernike descriptor was obtained for each lesion with 2,601 dimensions for Group *S_s_* and 15,876 for Group *S_l_*, based on Eqs 16, 18. The principal component analysis (PCA) was then used to reduce the large number of dimensions of the Zernike descriptors to reduce computation demand while minimizing information loss. To maintain 99.8% variance of the two lesion size groups, the number of principal component choices for the two groups are 80 and 68, respectively (Figure 5). The Zernike descriptors were projected on the selected principal components (eigenvectors), and the coefficients corresponding to these principal components were used for WMH3D shape clustering and classification.

#### WMH3D shape classification

A K-means algorithm was used for clustering and classification due to its simplicity and efficiency (Bharati et al., 2004). However, different initial seeds used in the clustering algorithm may generate different clustering results (Hamerly and Elkan, 2002). In this study, we randomly selected the initial clustering seeds from the shape lesion feature space and conducted 1,000 trials to assess the clustering results. We employed a gap statistic method to determine the optimal number of clusters for WMH3D shape clustering (Tibshirani et al., 2001).

1. The sum of the within-cluster dispersion *W_k_* is computed for each choice *k* clusters (*k* = 1, 2,…, *N*).

(21) $$W_{k}=\sum_{r=1}^{k}\sum_{x_{i}\in C_{r}}{\left(x_{i}-{\bar{x}}_{r}\right)}^{2}$$

where *x*_*i*_ is a data point, *C*_*r*_ denotes cluster *r*, and ${\bar{x}}_{r}$ is the vector mean of *C*_*r*_.

The *B* reference datasets is uniformly generated by randomly sampling from the bounding rectangle of the original dataset. By Eq. 21, $W_{k\,b}^{*}$ is computed for each *k* and *b* (*b* = 1, 2, …, *B*, *k* = 1, 2, …, *N*). Then, compute the estimated gap statistic


(22) $$G\,a\,p\,\left(k\right)=\frac{1}{B}\,\sum_{b}\text{log}\,\left(W_{k\,b}^{*}\right)-\text{log}\,\left(W_{k}\right)$$

2. let *l* = (1/*B*)∑_*b*_ log (*W*_*kb*_), compute the standard deviation

(23) $$s\,d_{k}={\left[\frac{1}{B}\,\sum_{b}{\left(\text{log}\,\left(W_{k\,b}^{*}-l\right)\right)}^{2}\right]}^{1/2}$$

Let $s_{k}=s\,d_{k}\,\sqrt{\left(1+1/B\right)}$. Choose the optimal number of shape clusters *via*


(24) $$\hat{k}=\text{smallest}\,k\,\mathrm{such}\,\mathrm{that}\,G\,a\,p\,\left(k\right)\geq G\,a\,p\,\left(k+1\right)-s_{k+1}$$

In gap statistic procedure, *N* is a pre-selected number of clusters such that $\hat{k}$ can be determined in the range of [1, *N*], and *B* is selected to calculate the value of *sd*_*k*_ in a statistical sense. In this study, *N* and *B* were set to 20 and 30, respectively.

For the size-normalized lesions, the feature dimension were 80 in Group **S_s_** and 68 in Group **S_l_**, the Gap values were calculated and displayed in Supplementary Figure 3; the optimal numbers of shape clusters were 5 for Group **S_s_** and 4 for Group **S_l_** according to Eq. 24.

**FIGURE 6:**
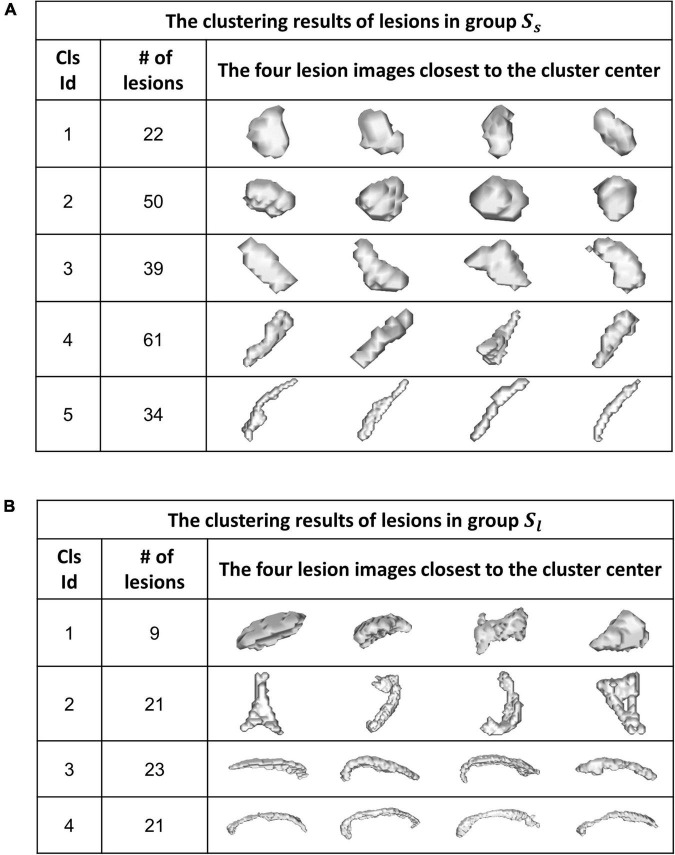
A K-means algorithm was performed to categorize WMH3D lesion shapes to different clusters for Group *S_s_* **(A)** and Group *S_l_* **(B)**.

Figure 6 shows the WMH3D shape clustering results using the K-means clustering algorithm (Hartigan and Wong, 1979) based on the cluster number of 5 for Group **S_s_** and 4 for Group **S_l_**. The second column shows the number of lesions in each shape cluster. The third column presents the four lesion images closest to their cluster means, as the representative lesion shapes corresponding to their clusters. All lesion images shown in the figure were normalized close to the voxel size of 80 for Group **S_l_** and 1500 for Group **S_l_**. The orientation-adjusted images can be seen in Figure 6 with the significant differences among the shape clusters.

### WMH3D texture feature and classification

#### Texture feature extraction

To obtain rotation-invariant texture features that are applicable to both small and large-size WMH3D, the fuzzy logic technique that we developed in (Gwo et al., 2019) was extended to WMH3D. Specifically, when segmenting WMH3D, false positives likely occurred at boundaries of a lesion, where signal intensity was usually low and thus led to biased estimation. To reduce chance of false positive, voxels with intensity at the lowest 1% were discarded. To reduce the variations of the signal intensity of individual subjects, a min-max normalization was applied to a WMH3D to normalize its voxel intensity based on Eq. 25.


(25) $$s\,\left(x,y,z\right)=\frac{f\,\left(x,y,z\right)-g\,M\,i\,n}{g\,M\,a\,x-g\,M\,i\,n}\,\mathrm{for}\,f\,\left(x,y,z\right)=\text{max}\,\left(\left(f\,\left(x,y,z\right),g\,M\,i\,n\right)\right)$$

where *f* (*x*,*y*,*z*) is the intensity of voxel *f* (*x*,*y*,*z*) and *s* ∈ [0,1], *gMax* = maximal voxel intensity of WMH3D and *gMin* = minimal voxel intensity of WMH3D.

For feature extraction, each voxel intensity was quantized into one of the *n* bins to create a histogram that represents voxel intensity distribution of a WMH3D. To minimize the interference of quantization to the frequency histogram, we used a fuzzy logic method (Gwo and Wei, 2013) to allocate normalized voxel intensity values to each of the pre-selected bins. Specifically, a normalized voxel intensity *s* was assigned proportionally two values, called fuzzy values, to the two neighboring bins according its relative positions to the bin centers (Supplementary Figure 4).

The fuzzy logic functions used for assigning voxels to the frequency histogram are presented in Eq. 26 (Gwo and Wei, 2013). The fuzzy value v[j] at bin j is calculated as:


(26) $$\left\{ \begin{array}{c} \begin{array}{c} v\,\left[0\right]=1\,\text{if}\,s\leq \frac{1}{2\,n}\\ \begin{array}{c} v\,\left[j-1\right]=\frac{2\,j+1}{2}-s\times n\\ v\,\left[j\right]=s\times n-\frac{2\,j-1}{2} \end{array}\}\,\text{if}\,\frac{1}{2\,n}\leq s\leq \frac{2\,j+1}{2\,n}\\ \begin{array}{c} v\,\left[j\right]=\frac{2\,j+3}{2}-s\times n\\ v\,\left[j+1\right]=s\times n-\frac{2\,j+1}{2} \end{array}\}\,\text{if}\,1-\frac{1}{2\,n}\geq s>\frac{2\,j+1}{2\,n}\\ v\,\left[n-1\right]=1\,\text{if}\,s\geq 1-\frac{1}{2\,n} \end{array} \end{array}\right.$$

where *n* = the total number of bins, and *j* = 0,…, *n*-1. Since the sizes of WMH3D lesions vary in a wide range (Figure 2), the WMH3D intensity frequency distribution histograms need to be further normalized before they can be compared. Herein, each histogram is normalized to have a total accumulative frequency of 1.

### WMH3D texture feature classification

Texture feature classification of individual WMH3D lesion images was conducted using a feature vector clustering method similar to those discussed above in the section of “WMH3D Shape Classification.” Of note, the texture feature vector was based on the frequency histogram presented above using the fuzzy logic method. The influences of different texture feature dimensions (i.e., the number of bins used to construct the intensity histogram) and the numbers of clusters on texture feature classification were explored using the same strategy discussed above for WMH3D shape feature clustering. The sum of within-cluster dispersion ***W*_*k*_** value was calculated with the cluster number from 2 to 20 and the feature dimensions from 2 to 15. As illustrated in Supplementary Figure 5, ***W*_*k*_** decreased with the increase of the cluster numbers. A noticeable “elbow” phenomenon was seen for a wide range of texture feature dimensions from 2 to 15. In (Hughes, 1968), there are two considerations in choosing an appropriate number of bins: (1) If the number of bins is too large, the fuzzy values accumulated in some bins become sparse, especially for small-sized lesions. Sparsity is problematic for any statistical analysis method. (2) Conversely, if the number of bins is too small, lesion features may not be effectively distinguishable. With these two reasons in mind, in this study, we selected ten bins for texture feature extraction.

The gap statistics discussed above was applied to determine the optimal number of texture feature cluster for pattern recognition based on the K-means algorithm for grouping (Hartigan and Wong, 1979). Supplementary Figure 6 shows that 5 is the optimal number of cluster.

### WMH3D potential growth index

Voxel intensity is an important feature in image analysis. In this study, the intensity information of voxels in the penumbra area was used to estimate the likelihood of lesion development. The intuitive assumption is that the higher the intensity value, the higher the probability that the lesion may develop. If the intensity of neighboring voxels (penumbra) around the identified lesions is within a reasonable range discussed below, these will be the voxels of interest for potential growth. The distance between the voxels and the boundary of the lesion should also be considered. It hypothesized that the farther the voxel is from the lesion boundary, the greater the contribution to the PGI.

**FIGURE 7:**
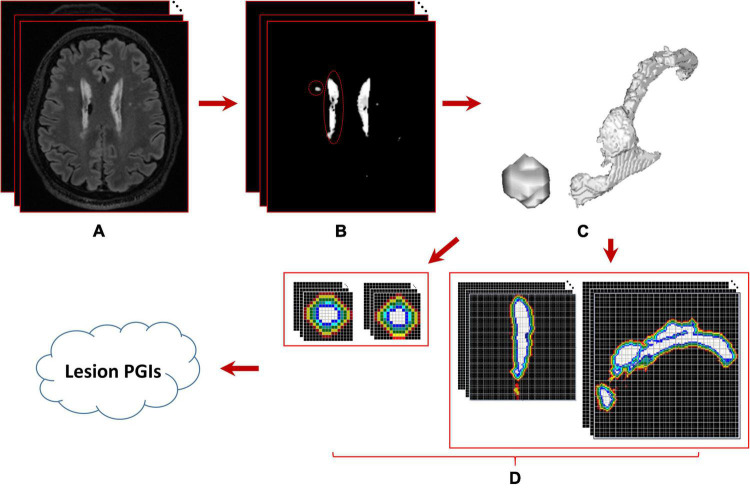
Schematic analysis pipeline for calculating WMH3D potential growth index (PGI). **(A)** The 3D image volume of a subject, **(B)** the results of WMH segmentation using the lesion prediction algorithm, **(C)** two enlarged WMH3Ds whose locations are marked with red circles in panels **(B,D)** the intensities of potential penumbra voxels within the range of interest are displayed in a lighter color with two different viewing directions. The sizes of these two 3D lesions are 91 and 12409 voxels, respectively.

For each subject, the interesting voxel set *P_w_* in lesion penumbra involves the calculation of PGI, and the corresponding intensity range is defined as follows:


(27) $$P_{w}=\{p\left(x,y,z\right)|m-f\left(x,y,z\right)\leq \gamma \times \sigma$$

where *f* (*x*, *y*, *z*) denotes the voxel *p*(*x*, *y*, *z*) intensity, *m* is the average intensity of all WMH3Ds in the subject, σ is the corresponding standard deviation, and γ is a user-defined positive real number. Dilation morphological operation is applied to mask image to iteratively generate *l* layers masks with one-voxel thickness surrounding the lesion, which is the interest area of the penumbra to estimate the PGI of the lesion. The schematic analysis pipeline for calculating WMH3D PGI is shown in Figure 7.

In this study, we chose γ = 2.5, which covers 99.38% of all WMH3D voxel intensities in a subject to demonstrate the presence of potential growth regions of WMH3D lesions. In the *l*-layer mask of the lesion, we are only interested in voxels with an intensity value greater than the value displayed by the red dotted line in the figure, which is *m*−2.5 × σ. These apparent layer masks are used to identify the relative location of a growth voxel. A growth voxel at an outer layers of these masks weights more in its contribution to the PGI. Specifically, the weight *w_i_* at *i*th layer, with total *l* layers, is given by the following equation:


(28) $$w_{i}=\frac{i}{\sum_{j=1}^{l}j}$$

Once the number of growth voxels at each layers were evaluated, PGI for each WMH3D lesion is calculated below:


(29) $$P\,G\,I=\frac{\sum_{i=1}^{l}G\,V_{i}\,w_{i}}{V_{l}}$$

where, *GV*_*i*_ = number of “growth voxels” found at the *i*th layer, and *V_l_* = the total number of voxels in all *l* layers for a WMH3D. All lesion images were evaluated for their PGIs with *l* set to 5. The PGIs estimated from the small lesions and the large lesions that are near the ventricle shown in Figure 7 are 0.0569 and 0.0844, respectively.

### The relationships between potential growth index and WMH3D shape and texture features

**FIGURE 8:**
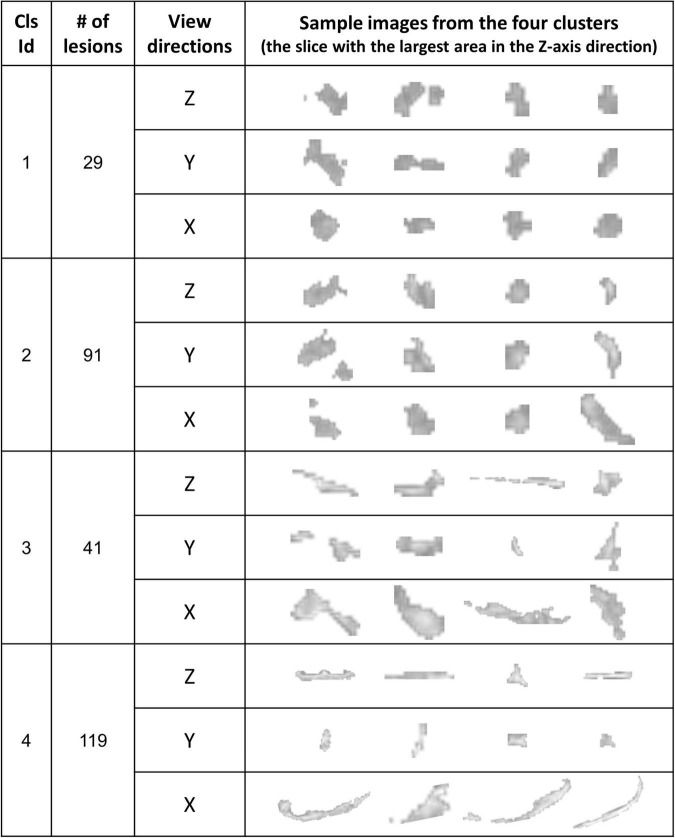
The WMH3D lesion images from the 32 subjects were classified into four clusters. The images at three view directions are displayed.

**FIGURE 9:**
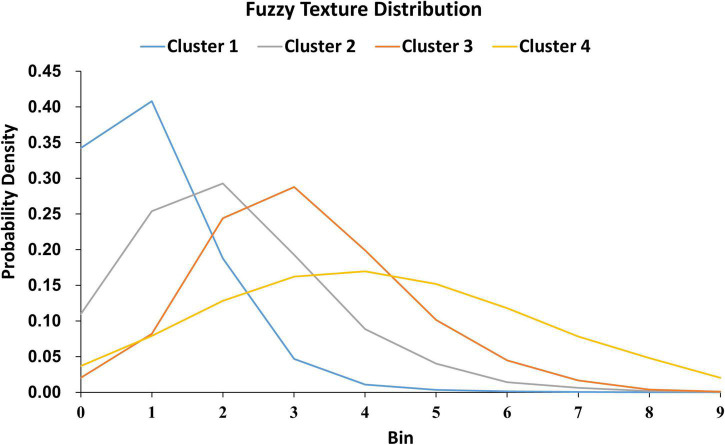
The 280 WMH3D lesion images from the 32 subjects were classified into four clusters based on their fuzzy image textures. The label on the y-axis is the probabilities of voxels in the lesion assigned to the bin.

For the shape and the texture clusters classified as shown in Figures 6, 8 above, one-way Analyses of Variance (ANOVAs) were performed to evaluate if there were significant differences in PGI among the shape or the texture clusters. Significant differences were found among both shape (*P* = 1.57 × 10^–3^ for Group *S_s_* and *P* = 3.14 × 10^–2^ for Group *S_l_* shown in Tables 1, 2) and texture (*P* = 1.79 × 10^–6^ shown in Table 3) clusters. Tables 4–6 also show the results after the Bonferroni corrections for multiple comparisons. As a reference, lesion volume size analyses are also included. It is worth noting that among the shape clusters in the *S_s_* group (Table 4 and Figure 6A), cluster 5 is significantly different from the other four clusters in terms of both PGI and lesion size. However, the PGI difference in cluster 5 from other four clusters was not likely driven by the lesion size because this cluster contains size evenly distributed between 30 and 250 voxels. For the *S_l_* group, significant PGI differences were only found between Clusters 1 and 4 and between Clusters 2 and 4 (Table 5 and Figure 6B). However, due to the large lesion size variance in each cluster, there was no significant difference in lesion size among the shape clusters (Table 2). Furthermore, significant PGI differences were found between cluster 4 and the other three clusters (Table 6). Of note, compared with the other three clusters, the average PGI value of cluster 4 is smaller and the texture color is lighter (e.g., high intensity) (Figures 8, 9).

**TABLE 1:** ANOVA analysis of the potential growth indices (PGIs) for shape clusters and corresponding lesion sizes in the *S_s_* group.

SHAPE (volume size ≤ 250 voxels)					
	Cluster 1	Cluster 2	Cluster 3	Cluster 4	Cluster 5
Number of lesions	22	50	39	61	34
PGI	0.0716 ± 0.0279	0.0667 ± 0.0362	0.0649 ± 0.0372	0.0577 ± 0.0312	0.0411 ± 0.0209
		Between-cluster difference: *P* = 1.5706 × 10^–3^, F = 4.5378			
Vol. size	60.3 ± 34.2	66.9 ± 41.5	75.7 ± 42.8	99.7 ± 61.7	128.7 ± 61.3
		Between-cluster difference: *P* = 1.1692 × 10^–7^, F = 10.3747			

**TABLE 2:** ANOVA analysis of the potential growth indices (PGIs) for shape clusters and corresponding lesion sizes in the *S_l_* group.

SHAPE (volume size > 250 voxels)				
	Cluster 1	Cluster 2	Cluster 3	Cluster 4
Number of lesions	9	21	23	21
PGI	0.0889 ± 0.0481	0.0674 ± 0.0299	0.0653 ± 0.0379	0.0494 ± 0.0213
Between-cluster difference: *P* = 3.1419 × 10^–2^, F = 3.1195				
Vol. size	502.1 ± 215.3	1482.9 ± 2605.8	2667.1 ± 3734.7	1295.3 ± 1466.2
Between-cluster difference: *P* = 0.1437, F = 1.8638				

**TABLE 3:** ANOVA analysis of the potential growth indices (PGIs) for texture clusters and corresponding lesion sizes for all 280 lesions.

Texture				
	Cluster 1	Cluster 2	Cluster 3	Cluster 4
Number of lesions	29	91	41	119
PGI	0.0783 ± 0.0289	0.0686 ± 0.0368	0.0668 ± 0.0327	0.0493 ± 0.0282
Between-cluster difference: *P* = 1.7865 × 10^–6^, F = 10.3463				
Vol. size	358.5 ± 1400.3	295.4 ± 636.9	500.7 ± 1738.5	733.6 ± 1821.7
Between-cluster difference: *P* = 0.2761, F = 1.2960				

**TABLE 4:** PGI and lesion size differences between shape clusters in the *S_s_* group.

Between clusters	PGI		Vol. size	
	F value	*P*	F value	*P*
Cluster 1 vs. Cluster 2	0.32	0.5715	0.42	0.5190
Cluster 1 vs. Cluster 3	0.54	0.4661	2.10	0.1530
Cluster 1 vs. Cluster 4	3.39	0.0693	8.03	5.82 × 10^–3^
Cluster 1 vs. Cluster 5	21.75	2.08 × 10^–5^**	22.66	1.49 × 10^–5^**
Cluster 2 vs. Cluster 3	0.05	0.8227	0.98	0.3255
Cluster 2 vs. Cluster 4	1.97	0.1631	10.33	1.72 × 10^–3^*
Cluster 2 vs. Cluster 5	13.82	3.67 × 10^–4^**	30.42	3.94 × 10^–7^**
Cluster 3 vs. Cluster 4	1.09	0.2980	4.49	3.66 × 10^–2^
Cluster 3 vs. Cluster 5	10.90	1.51 × 10^–3^*	18.65	5.00 × 10^–5^**
Cluster 4 vs. Cluster 5	7.67	6.77 × 10^–3^	4.83	3.04 × 10^–2^

Shown *P*-values are before Bonferroni corrections. To account for corrections, the thresholds are set at *P* < 5.0 × 10^–3^ to be considered significant (indicated by *) and *P* < 1.0 × 10^–3^ to be considered highly significant (indicated by **).

**TABLE 5:** Compare the PGI and lesion size between shape clusters in the *S_l_* group.

PGI between clusters	F value	*P*
Cluster 1 vs. Cluster 2	2.23	0.1466
Cluster 1 vs. Cluster 3	2.15	0.1527
Cluster 1 vs. Cluster 4	9.95	3.82 × 10^–3^*
Cluster 2 vs. Cluster 3	0.04	0.8382
Cluster 2 vs. Cluster 4	5.04	0.0304
Cluster 3 vs. Cluster 4	2.85	0.0986

Shown *P*-values are before Bonferroni corrections. To account for corrections, the significant threshold is set at *P* < 8.33 × 10^–3^ (indicated by *).

**TABLE 6:** Compare the PGI between texture clusters for all 280 lesions.

PGI between clusters	F value	*P*
Cluster 1 vs Cluster 2	1.70	0.1953
Cluster 1 vs Cluster 3	2.30	0.1338
Cluster 1 vs Cluster 4	24.51	2.02 × 10^–6^**
Cluster 2 vs Cluster 3	0.07	0.7947
Cluster 2 vs Cluster 4	18.55	2.55 × 10^–5^**
Cluster 3 vs Cluster 4	10.88	1.20 × 10^–3^**

Shown *P*-values are before Bonferroni corrections. To account for corrections, thresholds is set at *P* < 1.67 × 10^–3^ to be considered highly significant (indicated by **).

## Discussion and conclusion

In this study, we extended our prior work in WMH morphological analyses from 2D to 3D. A total of 280 3D lesions from 32 cognitively normal older adults with a volume size of greater than 30 voxels were used in shape and texture the analysis. When using Zernike transformation to extract shape features of 3D objects, volume normalization is a necessary process. In this regard, excessive scaling may enlarge the boundary details of small lesions while losing the details of large lesions. In this work, before clustering the shape of the lesions, WMH3D lesions were divided into two groups according to the size of the lesions to minimize volume normalization error. The texture features of the lesions used in this study were generated based on the intensity distribution. The fuzzy processing based on image intensity normalization for feature extraction reduced the influences of the intensity quantification. In addition, the intensity distribution was normalized by the size of the lesion, resulting in size independency.

The statistical data analyses showed that regardless of the volume size category of the lesions, PGI had significant differences among the shape clusters. The significant differences were also presented among the texture clusters. T_2_ FLAIR WMH lesions were mostly located around the ventricles. The lesions around the ventricle were usually longer in shape and had high voxel intensity in texture, and had lower average PGI values than lesions distant from the ventricles. These observations together suggest that WMH lesion anatomic locations, morphological characteristics, as well as the lesion texture may have impact on lesion progression. Further work with a large sample size and a longitudinal study design would allow us to address these clinically significant questions.

When the number of lesions is sufficient with a large sample size of subjects, the merging of different lesion size groups performed in the present study to reduce the influence of lesion size on the application of Zernike transformation would not be necessary. The etiology of WNH is complex and can be multifactorial (Alber et al., 2019). Given that healthy subjects and patients with hypertension were enrolled this study, we suspect, but cannot prove that WHM lesions observed likely reflect the presence of cerebral small vessel disease (Alber et al., 2019). Whether WMH3D shape and texture characteristics and location are related to different etiology also worth further studies. Finally, studies are also needed to optimize the algorithms and parameters of shape and texture feature extraction, clustering, and PGI estimation with a goal to apply this novel imaging processing method to clinical research.

## Data availability statement

The raw data supporting the conclusions of this article will be made available by the authors, without undue reservation.

## Ethics statement

The studies involving human participants were reviewed and approved by the Institutional Review Boards of the UT Southwestern Medical Center and Texas Health Presbyterian Hospital of Dallas. The patients/participants provided their written informed consent to participate in this study.

## Author contributions

C-YG conducted experiments, wrote code to analyze the data, interpreted the data, and wrote the manuscript. DZ prepared brain images and lesion segmentation. DZ and RZ interpreted the data, participated in the scientific discussions, and provided critical insights. All authors reviewed the manuscript and approved it for publication.
