# Supplementary material for: Brain white matter hyperintensity lesion characterization in 3D T2 fluid-attenuated inversion recovery magnetic resonance images: Shape, texture, and their correlations with potential growth
Source: Front Neurosci. 2022 Nov 24;16:1028929. doi: 10.3389/fnins.2022.1028929 (PMC9731131; doi:10.3389/fnins.2022.1028929)
Supplement: Supplementary file 1 [file Data_Sheet_1.pdf]

Supporting Information

Brain White Matter Hyperintensity Lesion Characterization in 3D T_2 Fluid-Attenuated Inversion Recovery Magnetic Resonance Images: Shape, Texture and Their Correlations with Potential Growth

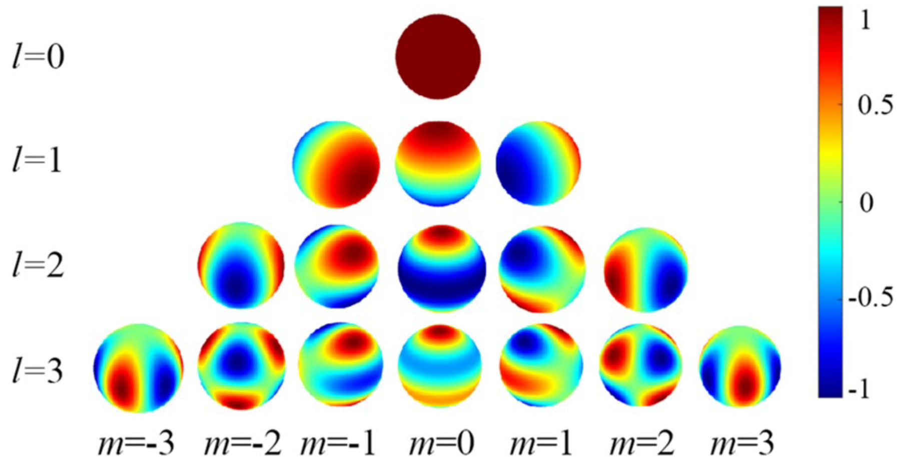

(a)

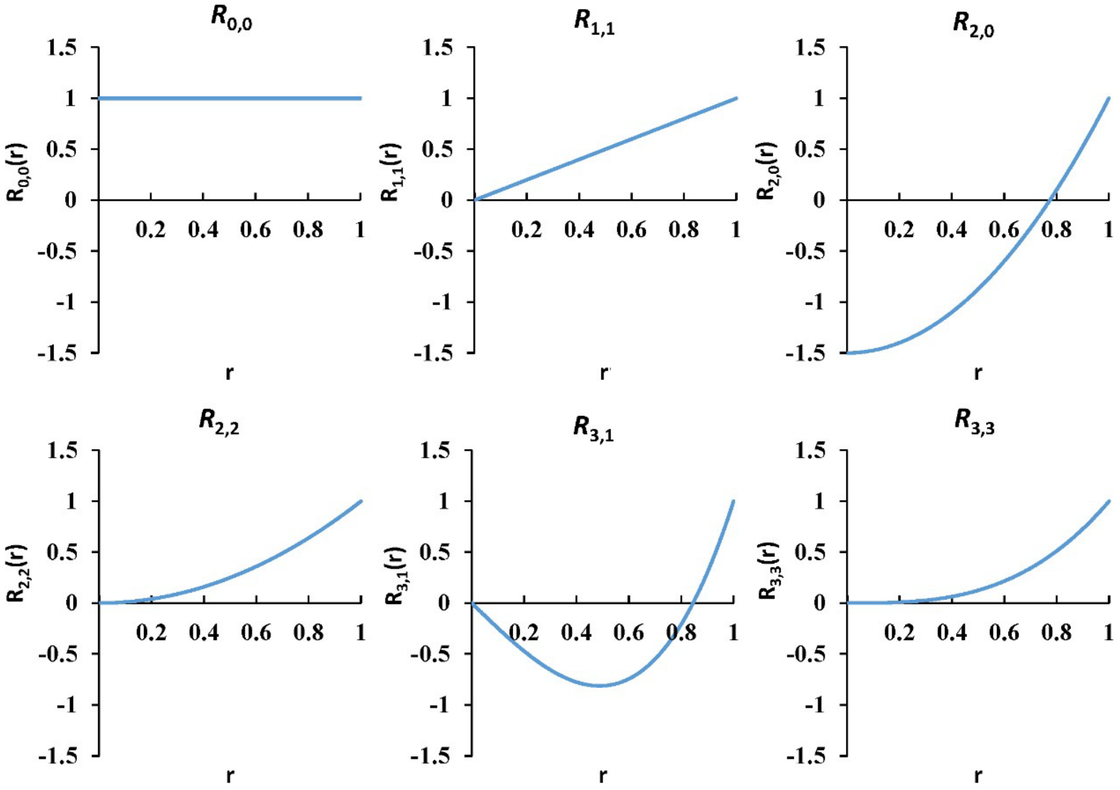

(b)

Figure S1. (a) The 3D spherical harmonics $YY^{mm}_{\ell}(\theta\theta, \phi\phi)$ of ll = 0, 1, 2, and 3 with order mm from - ll to ll are shown with the images from Liu, Huaping, Yong Fang, and Qinghua Huang. "Efficient representation of head-related transfer functions with combination of spherical harmonics and spherical wavelets." *IEEE Access* 7 (2019): 78214-78222. (b) The radial polynomials are illustrated.

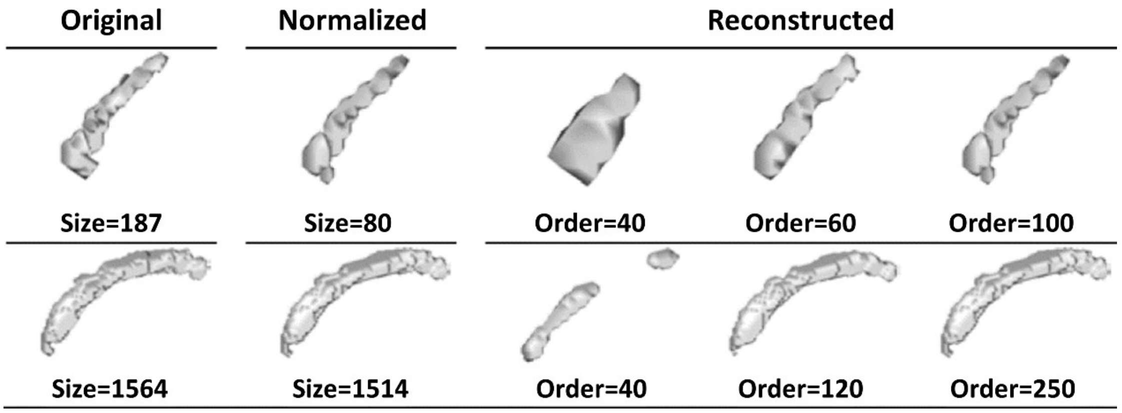

Figure S2. Zernike transformation 3D object reconstruction of two WMH3D lesions of different sizes at different orders.

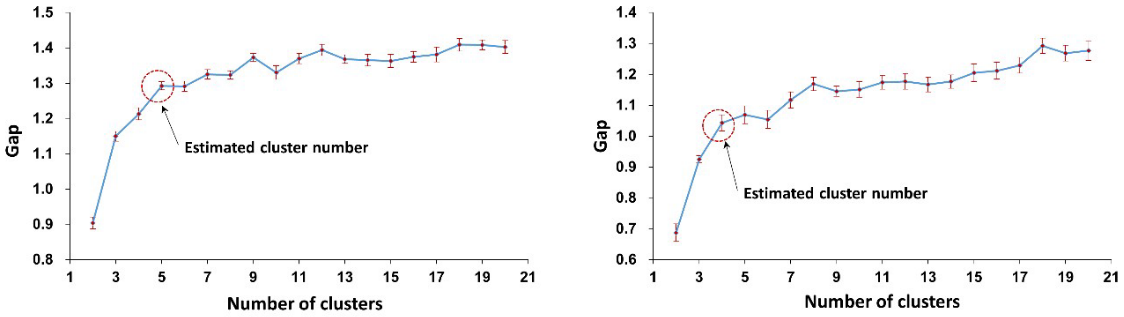

(a) (b)

Figure S3. The cluster number was chosen based on gap statistic where error bars were $\pm s_k$ for Group S_s (a) and Group S_l (b).

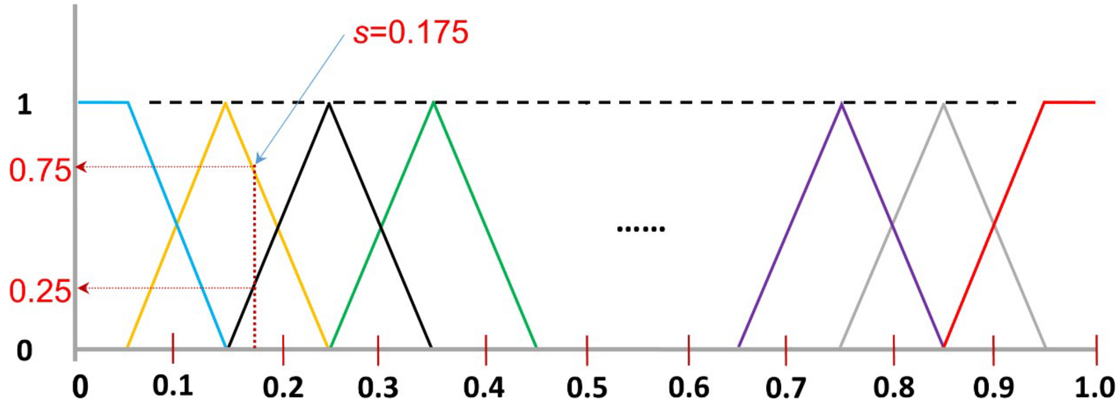

Figure S4. The fuzzy logic functions was used to assign voxels to ten bins : bin [0, 0.1] shown in blue, bin [0.1, 0.2] in orange, bin [0.2, 0.3] in black, bin [0.3, 0.4] in green, and bin [0.9, 1.0] in red. Normalized voxel intensity(s) was assigned to the two neighboring bins based on these assignments functions. For example, s of 0.175 was assigned to a frequency value of 0.75 to the bin [0.1, 0.2], and 0.25 to the bin [0.2, 0.3] as indicated by the vertical and horizontal dotted lines [72].

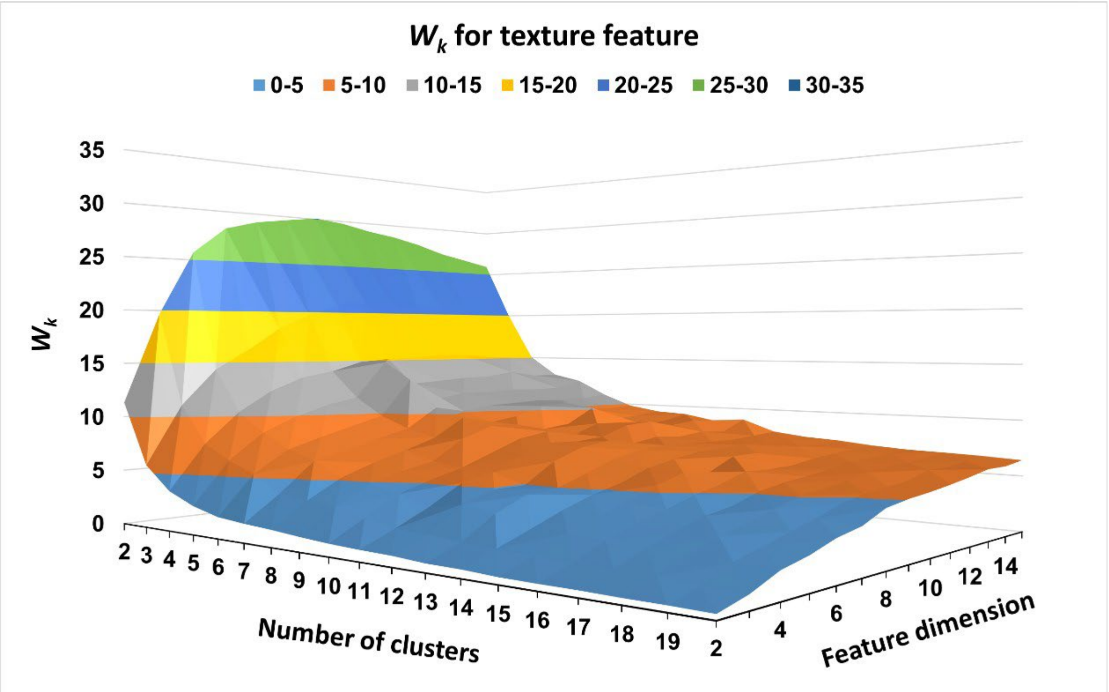

Figure S5. The within-cluster dispersion WW_{kk} as the function of texture feature dimensions and clusters dispalyed in each of the color bands . Note that a noticeable "elbow" phenomenon presents for a wide range of texture feature dimensions from 2 to 15.

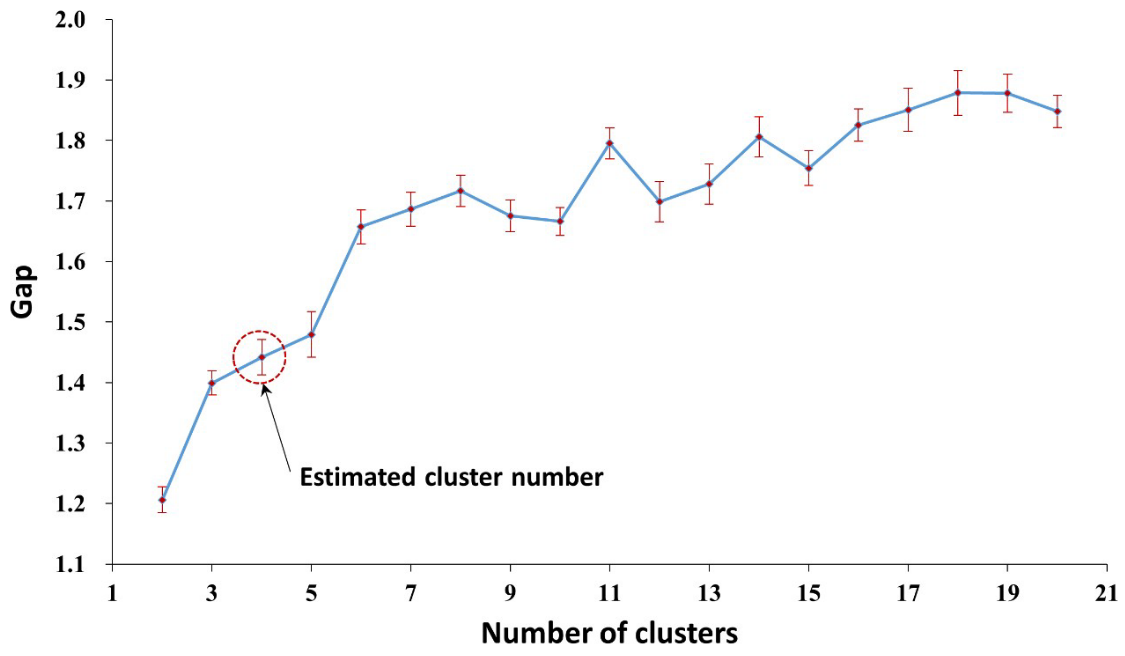

Figure S6. The estimated gap statistic Gap as a function of texture cluster number kk, error bars are $\pm ss_{kk}$.
